# The more health policies change, the more they change the same way

**DOI:** 10.1186/s13584-019-0308-6

**Published:** 2019-04-26

**Authors:** David Chinitz

**Affiliations:** Department of Health Policy and Management, School of Public Health, Hebrew University-Hadassah, POB 12272, 91120 Jerusalem, Israel

**Keywords:** Computer information technologies, Health reform

## Abstract

In a series of articles over the last 5 years, Richard Saltman, one of the foremost scholars in the field of comparative health systems has begun to question whether traditional pillars of these systems are in need of fundamental restructuring. In the wake of the financial crisis of 2008, Saltman argued for new modes of financing to cope with austerity, and re-examination of the concept of social solidarity. In a recent piece in this journal, he considers the challenges posed by the information revolution. This commentary raises questions regarding the particular impact of the information revolution as opposed to pressures that have beset health systems for several decades, and examines Saltman’s policy prescriptions in light of previous attempts to restructure health systems. It is suggested that whatever the path forward for health systems, failure to address the cultural gap between medicine as a profession and medical managerialism explains past reform shortcomings and is likely to hinder any restructuring responses to the information revolution.

“The more things change, the more they stay the same.” *Jean Baptiste Alphonse Karr,19th century*

“There is nothing new under the sun.” *Ecclesiastes*

“Things have never been this bad” *Rita Simon (paraphrased)*

“Things have never been better” *Steven Pinker (paraphrased)*

## Introduction

One could ask if health policy in most developed countries is going in circles, and whether there needs to be major structural change of most health systems? That is a question prompted by Richard Saltman [1], who proffers that the combination of fiscal austerity and the information revolution will call for significant redesign of health systems. I believe that what has been happening in most developed countries is a work in progress, though it seems that some key issues arise over and over again. We are not going in circles, but rather in spirals, coming back to old problems and dilemmas that keep reappearing, but with wisdom gained from previous attempts at solving them. Stakeholders and analysts continue to display varying opinions about the size of change in the health system and its environment and the relationship between the two. Sometimes health policy is seen as an ongoing process of social learning, and sometimes as major change. Depending on one’s view in this regard, policy prescriptions are also presented as significant shifts required to cope with big changes taking place in the environment, or, alternatively, as incremental adaptations.

Richard Saltman, who is a keen veteran observer and analyst of health systems, has, in recent years, published a number of pieces that reflect fairly major shifts in his own thinking about health systems. A fierce proponent of the position that most health reforms in Western countries have (and perhaps should have) avoided tampering with the dominantly public finance of their health systems, in 2015 Saltman began to advocate for various forms of public supplemental and private sources of finance [2]. In another piece he probed the limits of social solidarity in times of societal financial stress [3]. Now, in a piece published in this journal, Saltman argues that health systems will have to discover new, innovative ways of financing and delivering health services due to the shock of the information revolution and its social and economic impacts. For reasons not completely transparent, a point to be discussed below, the analysis is limited, purportedly, to tax funded European health systems. According to the article, the latter will have to confront three major disruptions: financial/political, clinical/medical, and organizational managerial. Following from these three categories, seven practical policy challenges are discussed.

This commentary will focus on the lessons and policies proposed by Saltman. First, we will take up the proposition that we are in the throes of a third industrial revolution spurred by computer-based information systems that has outsized impacts on health systems. Following this we will briefly relate to the focus of the paper on tax based national health systems. Then we will examine the three disruptions predicted for health systems. Finally, we will critically analyse the seven practical challenges that Saltman feels national health systems will have to cope with for the foreseeable future.

### We have all been here before

Saltman correctly reminds us that at least since the mid twentieth century governing health systems has been a delicate balancing act drawing on a “range of policy fields” to cope with “the concerns of sectoral interest groups.” For most Western European health systems this balancing act began in the first part of the twentieth century [4]. Negotiating with medical providers so that global national budgets would not explode was the order of the day for all governments seeking to provide universal coverage while limiting expenditure. The latter transformed into “cost containment” during the oil crisis of the 1970s and subsequent economic stagflation [5], and was accompanied by efforts to improve the responsiveness of publicly provided medical services in the 1980s [6]. Enter market mechanisms, whether public or private based [3, 6–8], total quality management, priority setting and evidence-based medicine. While all these health policy and management aspirations [9] left their marks on health systems [10], none of them individually, or any combination of them led to lower costs, better access, and higher quality – the so called “triple aim”- to any convincing degree. Choice for patients was expanded as part of these reforms [11] but at the same time, limits were put on private medical practice [12]. While the field of health policy and management spawned ideas that penetrated health delivery systems, all of the latter continue to seek “best practices,” and to identify high performing health systems and mimic them. The fact of the matter is that organizations such as the Mayo Clinic, Geisinger, and Inter Mountain, were identified as high performing (though not necessarily by any rigorous metrics and more by consensual reputation), and their roots lay further in the past and not solely a response to the demands for innovation that emerged in the last decades of the twentieth century [13].

Underlying the limited impact of reform efforts to date, is the gap that exists between front line medical professionals, and managers who seek to implement tools based on economic incentives, various forms of quality measurement, and even relate to organizational culture [14, 15]. While the latter is often discussed, there are clear indications that the managerial interventions of the last two decades have been orthogonal to the culture of medical practitioners [16, 17]. Recently, Kaiser Permanente has created its own medical school in order to close the gap between what is taught in medical school and what the organization feels is the orientation needed to practice medicine in its system. If anything, the information revolution as it has been enacted in health care has only widened the cultural gap [18].

Thus, the question that arises in connection with Saltman’s focus on the current information revolution is whether it will lead, necessarily according to Saltman, to fundamental changes that are significantly different from the reform efforts of the 1990s. Even more poignantly, if, as argued above, most of the fundamental changes proposed by health policy and management experts and implemented by national health systems have not radically changed the way medicine is practiced, controlled cost, reduced inequities, and increased quality, what are the prospects for innovations spurred by the information revolution? Indeed, Saltman himself points out that the computer revolution has led to small technical adjustments accompanied by a minor new funding allocation. Is the current environmental shock really a sea change of disruptions, and will the responses it evokes be very different from ideas touted in previous rounds of health reform?

### The focus on tax funded systems

Saltman’s focus on tax funded systems derives, perhaps, from an intuition that social insurance systems, which feature an intermediate, or “meso” level of governance in the form of statutory health insurance funds,^1^ already allow for varied sources of finance, such as supplemental insurance, and innovation. Regarding the latter, for example, health plans, such as those that exist in Israel, have proven to be centers for development of new projects aimed at quality improvement, coordination of care and efforts at reducing health inequities. Saltman appears to yearn for similar diversity in tax funded systems, and, indeed, refers to new financing initiatives in the Dutch social health insurance system and even to health system delivery initiatives in the largely private US system. In the Israeli case, at least, the health plans, which function in a contested market for insurees, seem to combine concern for both cost and quality, and certainly seek to improve their use of information technology in doing so. The question is posed whether creation of various forms of finance, allocation and delivery of health services at, say, the meso level of tax-based systems, does not lead, inevitably, to arrangements that begin to look like social health insurance systems.

### The three disruptions

#### Financial/political

Saltman, in concert with previous articles, sees the financial crisis of 2008 as creating a chronic condition of low economic growth that prevents governments from increasing finance for health systems. What is less clear in the piece is how exactly the computer information revolution will lead to less tax revenues for government. Is the idea that fewer workers will be needed due to replacement by technology? If wealth is being concentrated in large technological firms, will there not be any attempt to increase tax revenue from them? Later in the paper, Saltman refers to attempts to supplement health system funding by raising income taxes for that purpose, and in other places he has alluded to various forms of voluntary supplemental insurance plans. We will return to this below, but these instruments have been features of health financing arrangements for decades and are not coming onto the agenda because of the technological computer information revolution. The political moves and skills that will be needed to amplify the roles of these types of financing have been deployed in the past. It is possible that current fiscal conditions will raise the profile of alternative sources of finance for health systems, and perhaps the political processes that will be needed to tap them will evolve.

#### Clinical/medical

New information systems and technologies will, according to Saltman, call for an “upgrade” of existing medical services. Clearly, the digital way of doing things has made inroads into health and medical care delivery systems. In some tech-oriented cultures, such as in Israel, computers were placed on the desks of physicians in health plans and hospitals even before anyone knew what to do with them, and the surrounding organizations figured out how to integrate the new technologies [19]. On the other hand, in the US, despite massive investments, use of health information systems, upgrading the use of electronic health records has foundered on organizational, bureaucratic and cultural shoals, and problems of interoperability plague these efforts [20]. Where the impetus for technological adaptation stems from, whether it be the front line, the managerial level, or from government, appears to matter [21]. Avoiding an overly top down approach may be especially tricky in tax funded systems wherein central control of finance may run counter to decentralized efforts at technical/organizational innovation.

#### Organizational/managerial

The claim here is that organizational “stasis” will be out of step with the pace of technological change. The question is whether organizations that resisted or failed at concerted attempts at change due to previous external shocks will, out of lack of choice, rise to the occasion to cope with what are construed as even bigger shocks now. As already alluded to, the cultural gap between front line providers and managers, which has not been significantly reduced in most health systems, is likely to be the main obstacle to change as it has been in the past [22, 23].

### The seven practical policy challenges

1. Finding a more suitable balance between ethics and finance. Saltman refers to a number of signals, across different countries, that fiscal limitations, sometimes labelled “austerity”, are seriously hampering the ability of health systems to deliver on their promise of universally adequate care. Long queues for doctor visits, diagnostic procedures, and elective surgeries; limited access to life saving drugs and, in the UK, the near collapse of public emergency rooms have led governments to look for ways to consolidate management and for new sources of funds. For the first time governments are considering raising taxes to be earmarked for their health systems or demanding that their citizens take on more financial responsibility for their health care, including purchasing more quasi-public supplemental insurance (such as exists in Israel and France), or private commercial health insurance. National governments are seeking to offload some health services, such as elderly home care and nursing home services to local authorities. Notably, Saltman references the Netherlands in this regard, despite it being a social health insurance, and not taxed based system. This echoes the Adel Reform -that transferred responsibility for long term care from the health system to the municipalities -that took place in Sweden in the early 1990s, before the 2008 financial collapse or the computer information revolution. Cycles of centralizing, decentralizing and shifting responsibilities among various levels of government have persisted in health systems for decades. Whether or not the current fiscal and technological pressures will lead to a more stable allocation of responsibility and accountability across levels of government remains to be seen.
2. Develop better strategies to steer structural diversity. Saltman argues that health systems will have to do a better job of creating new organizational subsystems and networks, for example, public private partnerships, in order to allow for more room for flexibility and innovation. Again, these are not new ideas, but Saltman appears to suggest that the combination of austerity and technological change, must now catalyze the overcoming of obstacles that have hindered such reforms, to date. One wonders whether the institutional, bureaucratic and cultural barriers that have led to, at best, partial success of such endeavours in the past, will now be overcome due to a new sense of urgency deriving from the unprecedented conditions that Saltman claims are now confronting health systems.
3. Ensuring better coordination between health and social care. Health systems and analysts have identified the challenge of improving the “continuum of care” for a long time. Trying to create “under one administrative roof” arrangements and the need to discharge elderly patients from acute hospitals to community- based alternatives that include social care was a focus for integration of care and medical homes in the 1990s and early 2000s. Institutional barriers have not faded away in the face of these well-known needs.
4. Overcoming Institutional stasis. One can only agree with Saltman’s assessment that while examples of institutions that truly innovate can be found, they are the exception rather than the rule.
5. Integrating labor unions into change strategies. Clearly, any moves in the direction of diversity in organizational forms for health care delivery are likely to be restricted by unions, that are predicated on standardized, negotiated employment relations. This is pertinent, for example, to the Israeli situation, in which efforts to reward physicians for working full time in the public sector run afoul of linkages in wage negotiations in the economy, whereby salary increases for one sector create a precedent for the same in other sectors. Saltman suggests contractual financial shifts, such as having contracts that pay more to productive employees. But, reimbursement arrangements such as pay for performance, have had limited success [14]. Mintzberg [22] discusses the dangers of financial incentives in health care, but he does call for physicians to be made more cognizant of resource utilization and other managerial concerns without overriding the norms of medical profession. The question of how to get physician buy-in to new working arrangements is not new. The cultural gap between medical professionals and management [23] is most important here, and, indeed, new information technologies, especially when used, not solely to measure and reward performance, but also as a basis to improve communication about health delivery processes, might facilitate the type of union/management cooperation Saltman is alluding to.
6. Implementing patient centeredness. Saltman suggests that this notion is threatening to providers. Concepts such as patient choice, shared decision making, or putting the patient in the center, have been used to mask the real intent of patient centeredness which is to give patients more control over their care. How much control patients want over their care is open to discussion. But part of patient centeredness would appear to be the use of teams structured around each patient’s needs. This has also been discussed widely, but, given the actual structure of health care organizations, it remains an aspiration. Whether, as Saltman proposes, genetic code-based individualized care will challenge health systems to break down barriers among specialties and lead to more patient involvement remains to be seen. Ironically, personalized medicine based on genetic mapping may lead to deterministic medicine that makes patient involvement superfluous.
7. Incentivizing individuals to improve their own care. Saltman raises the spectre of a shift in orientation of tax based systems from collective to individual responsibility for health. He more than hints that it may be time to use negative incentives to improve individual health behaviors. Similarly, the behavioural economics movement touts “nudges” that will get people to adopt more healthy behaviors without coercion [24]. What is missing here, ironically, is the exploitation of the information revolution to develop new ways of informing people about healthy living, perhaps in combination with various forms of incentives. The information revolution, as much as it may stress health systems, also holds opportunities to make health systems more viable.

## Conclusion

Richard Saltman is one of the most astute and informed observers and analysts of European health systems. His concern for the future of tax based systems, in particular, is evident. Long a proponent of resisting alterations in the financing of tax based systems, and a passionate spokesperson for their solidaristic foundations, he has followed forthrightly the dictum that there comes a time to change one’s mind. Along with tapping public willingness, if such exists, to raise taxes to save public health systems - a matter arising now on the agenda in Israel - development of new, hopefully solidarity based and effectively regulated sources of additional finance, for example, collectively organized supplemental insurance, would appear to be merited. Many of the delivery system changes that Saltman discusses are, in his own words, not new ideas. Perhaps, however, they will be adopted more successfully in health systems due to both financial pressures and the threats and opportunities of the information revolution. An additional component, often referred to, but less reified and realized, is the creation of cultures of innovation, quality improvement, and efficiency, that can take advantage of some of the tools Saltman considers by bridging the gaps in orientation between medical and managerial professions. This may be the missing piece that, under the external pressures of austerity and technological innovation, can move health systems from spirals to more linear paths of improvement.

## Footnotes

1 Tax based, or Beveridgian health systems, are based on finance from general taxation, as in the United Kingdom. Social insurance, or Bismarkian systems, are based on statutory health insurance funds subject to rules linked to universal, or near universal coverage, as in Germany.
